# Associations between social support and poverty among older adults

**DOI:** 10.1186/s12877-023-04079-7

**Published:** 2023-06-23

**Authors:** Hui Liao, Sangsang Li, Dan Han, Mei Zhang, Jie Zhao, Yunyi Wu, Ying Ma, Chaoyang Yan, Jing Wang

**Affiliations:** 1 Department of Health Management, School of Medicine and Health Management, Tongji Medical College, Huazhong University of Science and Technology, Wuhan, Hubei China; 2 The Key Research Institute of Humanities and Social Science of Hubei Province, Huazhong University of Science and Technology, Wuhan, Hubei China; 3 Institute for Poverty Reduction and Development, Huazhong University of Science and Technology, Wuhan, Hubei China

**Keywords:** Social support, Health insurance, Family support, Poverty, Older adults

## Abstract

**Background:**

With population aging becoming a pressing global concern, social support is more meaningful for older adults. In particular, financial supports, such as health insurance and financial assistance derived from family, all play great role in assistance affairs. Research shows social support possibly has an impact on poverty, but the association between formal and informal supports is unclear. We are aimed at verifying the association between distinct social supports and exploring whether this association would affect poverty alleviation for older adults.

**Methods:**

A total of 2,683 individuals aged 60 years or older who have medical expenses were included in a survey conducted by the China Health and Retirement Longitudinal Study in 2018. A chi-square analysis and an independent samples T test all were used to explore the differences of social supports among old people with different economic condition. A binary logistic regression was aimed at analyzing the association between social supports and poverty for older adults. The structural equation model was established to evaluate the association between formal support and informal support and the mechanism(s) of social supports affecting poverty.

**Results:**

The overall average rate of reimbursement for outpatient care was 0.20 with standard deviation 0.22, and the average reimbursement rate of inpatient care for the poor older adults is nearly 5% lower than the average of the non-poor older adults. We found that having private health insurance and higher reimbursement rate of inpatient care were associated with lower likelihood of living in poverty for older adults. Formal support would directly affect poverty, but its impact on poverty through informal support is insignificant even if formal support is negatively associated with informal support.

**Conclusion:**

A dilemma in reducing the economic burden of disease and receiving family assistance for older adults was revealed, and a more complete health security and higher level of medical expenses compensation would be beneficial to prevent poverty. Optimizing the primary healthcare and increasing the percentage of insurance compensation, policies that focus on the specific cultural values and strengthening the role of supplementary insurance are advantaged for alleviating poverty among older adults.

## Introduction

### Social support and poverty

Social support has been defined as the assistance one person received from others on account of being cared about and respected by others [1]. According to the supplier, social support can be evaluated from both formal and informal aspects [2, 3]. Formal support refers to the assistance provided by formal organizations (i.e., government, institution, unit, and community) [4], while informal support comes from family members, other relatives, friends, neighbors, and co-workers who surround the individuals and vary in their closeness [5]. Meanwhile, social support encompasses distinct forms of assistance: instrumental (i.e., tangible) aid, financial assistance, emotional comfort, and provision of informational advice and appraisal [6].

In the contemporary times when population aging becomes a pressing global concern, social support is more meaningful for older adults. According to the United Nations report, the world population of older adults is growing with an annual rate of 2.6% [7], more than twice the growth rate of the whole population, and a consequence of the increasing percentage of older adults is a surge in the incidence and prevalence of age-related diseases [8]. On the one hand, serious disorders might lead to individual disability [9]; hence, receiving others’ assistance is important for older adults in their daily activities and healthcare. On the other hand, illness inflicts a heavy economic burden on older adults and their family through loss of income and increase in medical expenses [10], which could reflect the necessity for external financial support and social security. Moreover, older adults need emotional counseling if their poor health or social disconnectedness carries with it a risk of anxiety and depressive symptoms [11].

Social support derived from financial aspect plays a crucial role in assistance affairs. Social security policies and medical security systems are the primary means to resist adverse events and guarantee livelihood in formal support [3]. For instance, the American Medicaid Service for low-income residents and disadvantaged groups has covered 23.8% of the U.S. population by 2020 [12], and approximately 11% of the general policies of the 5th five-year economic, social, and cultural plan are directly addressing health-related issues with developing health insurance system and reducing out-of-pocket (OOP) expenditures for health services to 30% in Iran [13]. For the main informal support, previous studies indicate that family-based support is an important part of the lives of older adults who stay at home and in the community or village across the globe [14]. In addition, kin financial assistance is more necessary, especially for many older people lacking pensions and formal state services [15].

Being in or returning to poverty due to an illness is currently a pressing issue for social development, and social support could be effective buffers that struggle against the risk of poverty. Numerous studies confirmed that individuals or households receiving formal supports are less likely to be poor [16, 17]. Every 10% increase in American Medicaid coverage can reduce the risk of impoverishment by 8% for individuals [18], and Azeem MM et al. found that public transfer payments have a positive impact in reducing household poverty [17]. Meanwhile, financial assistance from kin and loaning from financial institutions could significantly resist economic hardships [19, 20]. However, the loan requirements of formal financial institutions are usually strict, and lacking credit could produce large amounts of debt. Compared with loaning, patients could transfer high health expenses to their offspring or other family networks, which could reduce the incidence of medical debt [21] and avoid a heavy economic burden. Verifying whether this association between social support and poverty consistently holds continues.

Despite a possible association between social support and poverty, little conclusive evidence exists on the mechanism(s) through which social support influences poverty among older adults, especially when an uncertain link exists between formal and informal supports. Opinions on the association between formal and informal supports are diverse. Some view that formal support will diminish informal support [22], whereas others regard both to be in a more frequently coexisting condition [23] or supplement mutually [24]. In fact, with the deterioration of health and weakening of social ability, obtaining coordinated social supports for older adults would be harder as they have a small scale on life and social intercourse [25]. Thus, doubts on the association of distinct social supports and whether this association would affect poverty alleviation for older people would persist.

Social supports may be heterogeneous among various sociodemographic factors. Previous studies suggested that access to social support is significantly related to gender. Older women tend to have greater social support than men [26], and older men are more reliant on spouses; conversely, women show a higher level of dependence on children and friends for financial and informational support [27]. For education, people with higher education might have a higher level of social support [28]. A study also observed a disparity in the specific quantities of households receiving public transfer payments between urban and rural residents [29]. Therefore, considering these confounding factors in exploring certain paths of different social supports affecting poverty among older adults is necessary.

### Social support in China

In China, formal supports are derived from social security policies and medical security systems. The social health insurance schemes in China have covered over 95% of the Chinese population, that is, nearly reaching the goal of universal coverage [30] and having reimbursed approximately half of their current medical expenditures [31]. These schemes consist of the Urban Employee Basic Medical Insurance (UEBMI; launched in 1998) and Urban and Rural Resident Basic Medical Insurance (URRBMI; launched in 2009) [32, 33]. Except for the above two insurance schemes, supplemental medical insurance and a government-funded Medical Care Aid program also exist to provide additional subsidies for residents with specific diseases in China to protect against disease risk and reduce the burden of disease for patients. Older adults could also receive services including healthcare, rehabilitation care, sports and entertainment activities, consultation, and life-long education services in various communities [34].

Informal supports are also an imperative part in Chinese society owing to traditional culture. Those associated with older adults and their families cover measures in aspects including financial support, life care, and spiritual comfort. Common financial supports include receiving expense transfer from family members or others, selling assets, loaning, and even gaining donations from charity [35, 36]. Especially in the Chinese countryside with strong humanity, raising funds in the private lending market is usually a zero-interest mutuality loan from relatives and friends for moral residents [37]. In addition, kin support is very important for the older people because of certain traditional Chinese culture, which emphasizes filial piety and complex interpersonal networks [38, 39]. Thus, the older people are often encouraged and taken care of by family members, relatives, neighbors, or friends [40].

The social support received by the older people under different economic conditions may vary in Chinese society. Non-poor households tend to purchase commercial medical insurance or long-term care services for the older people, whereas people living in economically disadvantaged households mostly benefit from monotonous informal support, especially for the rural empty-nest older people [41, 42]. Moreover, participating in some medical insurance might have unequal support effects for older people with different economic conditions, because the current insurance schemes are insufficient to address the inequity of access to healthcare [43, 44]. For instance, Xie X et al. using data from China find that richer people enjoyed a higher reimbursement rate for inpatient services (52.2%–58.7%) than their poorer counterparts (43.9%–44.1%) [44]. Therefore, exploring the distribution of social support received by older people under different economic conditions is meaningful.

In the context of implementing health poverty alleviation policies in China, scholars have explored the association between social support and poverty. Song ZJ et al. proposed that the supplementary medical insurance and the midrange commercial health insurance are the effective means to prevent poverty [45], but Xie MM et al. found that the effect may be weakened after the multiple supplementary medical insurances are superimposed [46]. Meanwhile, previous studies found that informal support could temporarily relieve the economic pressure and avoid a rapid falling to poverty, whereas some strategies such as selling assets and loaning may increase the economic vulnerability of families in the long term, particularly for large medical expenses [47]. Apparently, the association between social support and poverty remains unclear in China.

Moreover, the association between formal and informal supports is also complicated in Chinese society. Complementarity theory holds that both supports should cooperate and complement in function [48]. However, most households have difficulty accessing coordinated social supports that meet their needs, because most formal supports such as old age allowances and the minimum living standard security system, have high thresholds, small coverage, and low compensation levels. Empirical evidence from China has also shown that formal support may crowd out or crowd in informal support [3, 49]. The crowding-out effect refers that providing extra social security for older people will reduce intergenerational funds, whereas the crowding-in effect insists that increasing social security will encourage adult children to supply additional resources. Obviously, the association between both is important, and whether this association will affect the possible impact of social support on poverty is also uncertain in Chinese society.

Hence, the association between formal and informal supports as well as the influencing mechanism of this association on poverty for older adults is not clear enough. The present study intends to focus on the health insurances and kin financial support to achieve the following purposes: (1) compare the distribution of social support of the older adults in different economic conditions, (2) examine the association between different social supports and poverty among older adults, and (3) explore the influencing paths among formal support, informal support, and poverty in a sample of older adults aged 60 and over in China.

This study contributes by recommending a better coordinated social support system with added focus on preventing health-economic risk of older adults. Moreover, knowing the association among health insurance, family supports, and poverty clearly has profound relevance in building a harmonious kinship, maintaining individual wellbeing, and spurring socio-economic development, which also has important policy implications in a society with high poverty across the world.

## Materials and methods

### Data and sample

The present study used cross-sectional data extracted from the China Health and Retirement Longitudinal Study (CHARLS) in 2018. CHARLS is a longitudinal survey conducted by the National School of Development at Peking University, aimed to be representative of the Chinese residents aged 45 years and older and their spouses. The national baseline survey of CHALRS was conducted since 2011, and a follow-up survey will be conducted every two to three years thereafter. Thus far, CHARLS has fulfilled regular surveys in 2011, 2013, 2015, and 2018. In theory, the 2018 data are the most recent available, using 2018 data is more likely to reflect phenomenon that are at the forefront of research subject and closest to the present than any other period of data. Moreover, the main questionnaire includes information on basic demographics, family structure and financial support, health status, healthcare and health insurance, employment, and household economy (income, consumption, and wealth).

The regular survey adopts multi-stage sampling. First, 150 county-level units (counties or urban districts) were randomly chosen proportional to population size from 30 provincial administrative units across the country (excluding Tibet, Taiwan, Hong Kong, and Macao). Then, three villages or communities are randomly selected as primary sampling units from each county-level unit using the Probability Proportional to Size (PPS) sampling, for a total of 450 villages/communities. The sample in 2018 surveys comprised 19,817 individuals from 12,400 households, and a total of 7,739 individuals aged 60 years or older with medical expenses retained. Among the remaining 7,739 individuals, 4,778 contained missing values in variables of interests, such as whether they received financial support from their children or relatives and friends, and 278 contained unusual values regarding OOP medical expenses and total medical expenses. Afterhandling the missing and unusual data, 2,683 samples were remained in the present study.

### Variables

#### Dependent variable

The dependent variable is poverty, and the data were obtained from the Harmonized CHARLS, which is a user-friendly version of a subset of the CHARLS interviews. Harmonized CHARLS was created by the USC Gateway to Global Aging Data team to improve accessibility of data to researchers and subsequently facilitate comparisons among different waves. Poverty was measured by the annual household income per capita according to the total household income and the total family population in 2018. The official poverty line in China is the 2011 per capita net income of 2,300CNY. After the Consumer Price Index adjustment, it is converted to 2,649.6CNY in 2018. Then, the Chinese currency RMB is converted into U.S. Dollars on the basis of the yearly average exchange rate in 2018 (1USD = 6.62CNY) according to the Bank of China. Therefore, people living in households with annual income per capita that fell below 400.24USD were coded as poor in this study.

The total household income and the total family population are directly obtained from the Harmonized CHARLS database, where the total household income is the sum of all income at the household level, including earning income, capital income, pension income, income from government transfers, other sources of income, and the total income from other household members. The total family population includes the respondents, spouses, parents (including biological parents, stepparents, and adoptive parents), parents-in-law, children, siblings, siblings-in-law, and other household members.

#### Independent variables

This study categorized social support as formal and informal supports, which were both assumed as the independent variables. The data about formal support come from the healthcare and insurance section of CHARLS 2018, and the data of informal support come from the time transfer and transfers section of CHARLS 2018.

Formal support was examined in the of public health insurance, private health insurance, reimbursement rate of inpatient care, and reimbursement rate of outpatient care. Among them, public or private health insurance was measured by asking the type of health insurance that respondents hold. The responses, including UEMI, URRBMI, and Medical aid, are regarded as having public health insurance, whereas private health insurance includes types purchased by work unit or individual. Both reflected the breadth of formal support, and both dichotomous variables were created and coded as 1 “having public health insurance” or “having private health insurance” and 0 “not having public health insurance” or “not having private health insurance”. Reimbursement rate of inpatient care, which was the ratio of the total hospital expenses minus the hospital OOP expenses to the total hospital expenses and reflected the degree of formal support, was the same with the calculation of the reimbursement rate of outpatient care. These ratios range from 0 to 1.

Informal support includes three indicators: financial support from children/grandchildren, financial support from relatives or others, and total family financial support. Whether respondents had received financial support was judged by asking them the amount of cash gifts or payment of bills they received from their children/grandchildren or from relatives and friends, except for parents/children/siblings, respectively. They reflected the breadth of informal support. Financial support from children/grandchildren or that from relatives or others was coded as 1 “having received” if the amount was more than RMB 0; otherwise, 0 “No” if participants did not receive any financial support. Then, the amount of cash received was summed up and divided into three ranges: RMB 0 is coded as “0”; RMB 1–6,700 was coded as “1”; and over RMB 6,700 was coded as “2”, forming the indicator of total family financial support, which reflected the degree of informal support.

#### Control variables

To account for observable potential confounding variables, control variables included in the following models were gender, age, marital status, education level, region, and self-rated health status (see Table 1). Self-rated health was measured by asking the interviewees how they felt in terms of their general state of health, the responses ranged from “very good” to “very poor”. This item was one of the widely used validated indicators of health in the field of social sciences. All the control variables were from the demographic backgrounds, family, and health status part of CHARLS in 2018.

**Table 1:** Code and question description of variables

Variables	Code	Question Description
Age	0 = 60–75, 1 = > 75	What is your actual date of birth
Gender	0 = male, 1 = female	Interviewer recorded R’s gender
Education	0 = illiterate, 1 = elementary school and below, 2 = middle school, 3 = high school and above	What is the highest level of education you have attained now (not including adult education)?
Marital status	0 = married, 1 = unmarried (divorced, widowed, never married)	What is your marital status?
Region	0 = urban community, 1 = rural village	Was the type of address village or city/town?
Self-rated health status	0 = good (very good, good), 1 = fair, 2 = poor (very poor, poor)	Would you say your health is very good, good, fair, poor or very poor?
Public health insurance	0 = no, 1 = yes	Are you the policy holder/primary beneficiary of any of the types of health insurance listed below? (circle all that apply)
Private health insurance	0 = no, 1 = yes	Are you the policy holder/primary beneficiary of any of the types of health insurance listed below? (circle all that apply)
Reimbursement rate of inpatient care	0–1	
Reimbursement rate of outpatient care	0–1	
Financial support from children/grandchildren	0 = no, 1 = yes	During last year, what’s the amount of financial support received from [XChildName] when he/she/they was/were not living with you?
Financial support from relatives or others	0 = no, 1 = yes	During last year, what’s the amount of financial support received from [name of sibling]? During last year, did you ever receive cash gifts/other financial support from any other relatives or friends except for parents/ children/ siblings? Or any in-kind giving?
Total family financial support	0 = 0 CNY, 1 = 1–6700 CNY, 2 = > 6700 CNY	
Poverty	0 = non-poor, 1 = poor	

### Statistical analysis

First, frequencies and cross-tabulations provided the distribution of socio-demographic variables, formal support, and informal support variables. Meanwhile, chi-square analysis was employed to explore whether different social supports or socio-demographic conditions have significant differences between non-poor and poor old people.

Then, the study used an independent samples T test to explore the differences in the reimbursement rate of inpatient care or outpatient care between non-poor and poor old people. Means and standard deviations showed a degree of concentration and dispersion of reimbursement rate in older adults.

Next, the study used a binary logistic regression to analyze the association between social supports and poverty for older adults under controlling sociodemographic variables and examine whether different social supports have impacts on poverty. If a significant impact exists, we judge the change direction of the association between the certain support and poverty through its corresponding coefficient.

Finally, a structural equation model (SEM) was established to evaluate the association between formal and informal supports and draw the paths of social supports influencing poverty by defining the formal and informal supports as two latent endogenous variables, sociodemographic and health-related information as latent exogenous variable, and poverty as observed endogenous variable. In this SEM analysis, the maximum likelihood method estimation was used.

For the SEM, the *χ2* statistic is usually significant in large sample studies, often causing researchers to reject appropriate models that should be accepted [50]. Therefore, this study used other fit indices, including approximate root mean square error of approximation (RMSEA), Akaike’s information criterion (AIC), and the expected cross-validation index (ECVI), the adjusted goodness of fit index (AGFI), comparative fit index (CFI), and Tucker-Lewis index (TLI). A good model fit is achieved if the GFI and AGFI values are above 0.90, CFI and TLI values are above 0.95, and the RMSEA value is below 0.05, providing a reasonable and appropriate fit [51]. Meanwhile, the AIC and ECVI values of the default model to be below those of independent and saturated models would be better.

The structural equation model was applied by AMOS 23.0. Additionally, the Chi-square analysis, independent samples T test, and logistic regression were executed using SPSS 12.0, with statistical significance at *P* < 0.05.

## Result

Table 2 summarizes survey participants’ characteristics and showed poverty incidence difference within subgroups of social supports or control variables. In general, the average age was 69.34 with standard deviation 6.72, and 80.4% were between 60–75 years old. The respondents comprised 1,361 females, accounting for approximately 50.7% of the total sample. Approximately 29.3%, 44.3%, and 16.0% of the respondents were illiterate, with elementary or middle school education, separately. Only 10.4% had high school education and above. Most were married (79.4%) and lived in a rural village (58.2%). Regarding health, 47.2% reported poor health. The majority of respondents had public health insurance (97.4%), but only 1.7% purchased private health insurance. Older adults mostly received financial support from children/grandchildren (84.1%), whereas only 30% received it from relatives or others, and more than half had annual total family financial support between RMB 1–6,700 (59.0%).

**Table 2:** Sample characteristics and the poverty incidence comparison within subgroups of social supports or control variables

Item			Total	Poverty (n/%)		*χ2*	*P*
			(n/%)	Non-poor	poor		
Control variables		Age				10.756	< 0.01
		60–75	2158(80.4)	1709(79.2)	449(20.8)		
		> 75	525(19.6)	381(72.6)	144(27.4)		
		Gender				0.200	0.654
		male	1322(49.3)	1025(77.5)	297(22.5)		
		female	1361(50.7)	1065(78.3)	296(21.7)		
		Education level				68.817	< 0.001
		illiterate	786(29.3)	563(71.6)	223(28.4)		
		elementary school and below	1188(44.3)	904(76.1)	284(23.9)		
		middle school	429(16.0)	362(84.4)	67(15.6)		
		high school and above	280(10.4)	261(93.2)	19(6.8)		
		Marital status				12.728	< 0.001
		married	2131(79.4)	1691(79.4)	440(20.6)		
		unmarried	552(20.6)	399(72.3)	153(27.7)		
		Region				123.858	< 0.001
		urban community	1122(41.8)	992(88.4)	454(11.6)		
		rural village	1561(58.2)	1098(70.3)	1669(29.7)		
		Self-rated health status				13.707	< 0.01
		good	280(10.4)	232(82.9)	48(17.1)		
		fair	1137(42.4)	910(80.0)	227(20.0)		
		poor	1266(47.2)	948(74.9)	318(25.1)		
Independent variables	Formal support	Public health insurance				2.604	0.107
		no	70(2.6)	49(70.0)	21(30.0)		
		yes	2613(97.4)	2041(78.1)	572(21.9)		
		Private health insurance				10.506	< 0.01
		no	2638(98.3)	2046(77.6)	592(22.4)		
		yes	45(1.7)	44(97.8)	1(2.2)		
	Informal support	Financial support from children/grandchildren				10.417	< 0.01
		no	427(15.9)	358(83.8)	69(16.2)		
		yes	2256(84.1)	1732(76.8)	524(23.2)		
		Financial support from relatives or others				0.821	0.365
		no	1878(70.0)	1454(77.4)	424(22.6)		
		yes	805(30.0)	636(79.0)	169(21.0)		
		Total family financial support				13.197	< 0.01
		0CNY	332(12.4)	277(83.4)	219(16.6)		
		1-6700CNY	1582(59.0)	1196(75.6)	1285(24.4)		
		> 6700CNY	769(28.7)	617(80.2)	619(19.8)		

For the control variables, older adult poverty was significantly related to age, education, marital status, region, and self-rated health status (*P* < 0.01). Higher poverty incidence occurs in the groups of older people over 75-year-old, illiterate, unmarried, living in rural village, and within poor health condition.

For the social supports, older adult’ poverty was significantly related to private health insurance, financial support from children/grandchildren, and total family financial support. Compared with older adults who held private health insurance or did not receive financial support from children/grandchildren, poverty incidence for people without private health insurance or receive financial support from children/grandchildren was higher. In addition, poverty incidence in the population with family financial support range of RMB 1–6,700 was higher than those who did not receive any family financial support and whose amount was larger than RMB 6,700.

Table 3 shows the values of reimbursement rate of inpatient care and that of outpatient care in older adults being non-poor and poor. In total, the average reimbursement rate of inpatient care was 0.49 with standard deviation 0.22, and that of outpatient care was 0.20 ± 0.22. An independent samples T test showed significant differences in the reimbursement rate of inpatient care of older adults among different economic conditions (t = 5.289, *P* < 0.001). Specifically, the average reimbursement rate of inpatient care for the poor older adults is significantly lower than that of overall samples and nearly 5% lower than the average of the non-poor older adults. These findings suggested the reimbursement rate of outpatient care for older adults is low, and the compensation for hospital expenses of the poor older adults is insufficient.

**Table 3:** Reimbursement level of inpatient care and reimbursement level of outpatient care by poverty

Variable		Poverty (Mean ± SD)		t	*P*
		Non-poor (*n* = 2090)	Poor (*n* = 593)		
Formal support	Reimbursement rate of inpatient care	0.50 ± 0.22	0.45 ± 0.24	5.289	< 0.001
	Reimbursement rate of outpatient care	0.20 ± 0.22	0.19 ± 0.19	1.900	0.058

Table 4 examines the association between social supports and poverty. Only private health insurance and reimbursement rate of inpatient care were statistically significant (*P* < 0 0.05). Private health insurance and reimbursement rate of inpatient care were protective factors for its OR of < 1. In other words, having private health insurance and higher reimbursement rate of inpatient care were associated with lower likelihood to be living in poverty after controlling for sociodemographic factor. This finding indicates that purchasing private health insurance and increasing the reimbursement rate of inpatient care could be helpful for preventing economic risks.

**Table 4:** Associations between social supports and poverty among older adults

Variables		OR	95% CI
Public health insurance (Ref no)	yes	0.779	0.447–1.355
Private health insurance (Ref no)	yes	0.094*	0.013–0.698
Reimbursement rate of inpatient care		0.457***	0.300–0.697
Reimbursement rate of outpatient care		0.938	0.585–1.505
Financial support from children/grandchildren (Ref no)	yes	1.162	0.623–2.165
Financial support from relatives or others (Ref no)	yes	0.867	0.691–1.088
Total family financial support (Ref 0CNY)	1-6700CNY	1.198	0.597–2.404
	> 6700CNY	1.034	0.496–2.155
Age (Ref 60–75)	> 75	1.300*	1.022–1.654
Gender (Ref male)	female	0.788*	0.637–0.975
Education (Ref illiterate)	elementary school and below	0.901	0.718–1.132
	middle school	0.639**	0.457–0.895
	high school and above	0.278***	0.166–0.468
Marital status (Ref married)	unmarried	1.311*	1.034–1.663
Region (Ref urban community)	rural village	2.599***	2.077–3.253
Self-rated health status (Ref good)	fair	1.297	0.907–1.854
	poor	1.479*	1.042–2.099
Constant		0.212***	

OR (odds ratio) stands for the relative risk, the independent variable is a risk factor if OR > 1 or a protective factor if OR < 1. 95% CI (95% Confidence Interval) denotes the confidence interval estimates the range of the OR-value with confidence level of 95%.

* *P* < 0.05, **P < 0.01, ***P < 0.001

It also indicated that poverty was associated with age, gender, education, marital status, region, and self-rated health status (*P* < 0 0.05). Advanced age and unmarried, living in rural area, and poor health were associated with greater likelihood of falling into poverty (OR > 1); whereas people being female and with higher education were less likely to be poor (OR < 1). These results reflect that the economic risk for the older men with low education, unmarried, living in rural areas, and with poor health would be greater.

**Fig. 1:**
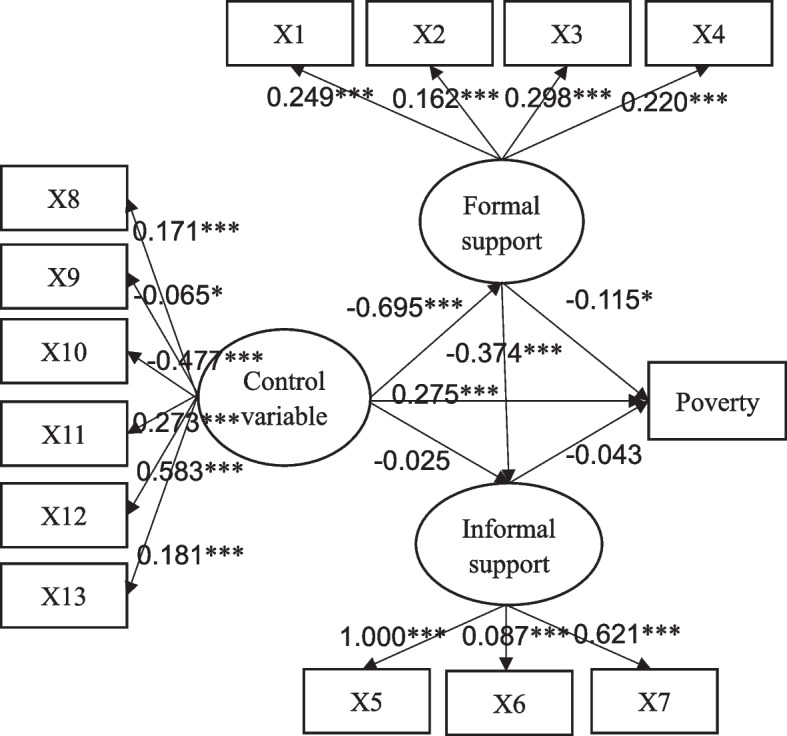
The path diagram and standardized estimate in SEM. Note: X1-X4 are public health insurance, private health insurance, reimbursement rate of inpatient care, reimbursement rate of outpatient care, successively. X5-X7 are financial support from children/grandchildren, financial support from relatives or others, total family financial support, successively. X8-X13 are age, gender, education, marital status, region, self-rated health status, successively. * *P* < 0.05, **P < 0.01, ***P < 0.001

Figure 1 explores the influencing mechanisms of formal support, informal support, and confounding factors on poverty among older adults and showed the results of SEM. The model fitting results of the SEM were all reasonable (see Table 5). Older adults having public or private health insurance, gaining higher reimbursement rate of inpatient care or outpatient care would be within stronger formal support (*P* < 0.001). Similarly, older adults who received financial support from children/grandchildren or other relatives and friends or whose amount of family financial transfers received was larger was more likely to possess stronger informal support (*P* < 0.001). Furthermore, the effects of reimbursement rate of inpatient care and financial support from children/grandchildren were the most prominent, respectively, which illustrated that the support from adult children and degree of hospital expense compensation as strong determinants of social support for older adults.

**Table 5:** Model fit indices for the structural equation modeling on formal support, informal support and poverty

		Lack of fit index			Goodness of fit index		Incremental fit index	
		RMSEA	AIC	ECVI	GFI	AGFI	TLI	CFI
Model 1	Default model	0.011	175.572	0.065	0.997	0.993	0.991	0.995
	Saturated model		210.000	0.078				
	Independence model		3153.725	1.176				

The default model is the hypothetical model established by this study; the saturated model takes into account the associations between all variables; the independent model does not include association between any variables

The central paths in Fig. 1 showed that formal support has a significant effect on informal support. With its path coefficient at -0.374, it indicates a negative association between both. It indicated that a “crowding out effect” is possible for formal to informal support, that is, family members and other relatives or friends would provide less financial support to older adults after they participate or have stronger social security.

Table 6 presents various effect values of different social supports on poverty from the SEM. Under controlling confounding factors, the direct and indirect effects of formal support on poverty are all significant (*P* < 0.05). Although the directions of its direct and indirect effects are opposite, the total effect value is -0.099, which illustrated that older adults are less likely to be poor with more powerful formal support. However, the effects of informal support on poverty among older adults were statistically insignificant (*P* > 0.05). Hence, the impact of social support on older adults’ poverty is manifested in the direct role of formal support.

**Table 6:** The standardized effects of formal support and informal support on poverty

Path	Direct effect	Indirect effect	Total effect
Formal support-Poverty	-0.115*	0.016***	-0.099***
Informal support-Poverty	-0.043	—	-0.043
Control variable-Poverty	0.275***	0.070***	0.345***

* *P* < 0.05, **P < 0.01, ***P < 0.001

## Discussion

This study found that social support received by non-poor and poor older adults has an imbalance. Especially, the average reimbursement rate of inpatient care for the poor older adults is significantly lower than that of the non-poor older adults, and the overall average rate of reimbursement for outpatient visit is low. Then, formal supports are associated with poverty among older adults, who own private health insurances, or those with a higher reimbursement rate of inpatient care will be less likely to be poor. Last but not least, formal support is negatively associated with informal support, and positive formal support would directly be helpful for poverty alleviation. Nonetheless, its impact on poverty through informal support is insignificant. The possible explanations for these results could be related with the imbalance in health services utilization, specific cultural concepts, and decrease in OOP medical expenses.

The imbalance in health services utilization possibly encourages the formation of inadequate compensation for health expenses, particularly for those poor older adults. First, individuals tend to be hospitalized in tertiary hospitals, as Chinese community-based healthcare delivery and primary care are limited in medical resources, and inpatient service is inevitable for individuals suffering from serious illnesses [52, 53], which will entail substantial cost. Thus, the high cost of hospitalization makes it impossible for social health insurance to increase the percentage of outpatient compensation. Second, most insurance plans only cover inpatient care cost or set a high deductible level for outpatient care cost. In this case, older adults, who have resource scarcity, intend to endure “minor” illnesses and not seek outpatient treatment to avoid medical expenses [53]; consequently, this scenario triggers a cycle between low reimbursement rate of outpatient visit and inadequate outpatient services utilization. Third, poor older adults and their families with low level of education may have to quit the medical expense compensation due to their confusion on the complex reimbursement regulations of healthcare, and studies have found that lower-educated individuals or families are usually in worse economic conditions [54].

Possibly, the negative association between formal support and informal support could be produced under specific cultural values. This finding is similar to that of Nikolov P et al. [55], which has also demonstrated that social security benefits lower the propensity of adult children to transfer income to older parents; that is, a “crowding out effect” may occur for formal support on informal support. On the one hand, this phenomenon may be influenced by the changing of traditional family value under the constraints of reality. Traditionally, the younger generation are expected to show respect and filial piety and provide support to senior members to help them enjoy old age [56]. However, constrained by limited resources, increasing financial support for older adults will inevitably reduce the investment for offspring education and daily consumption [3]. Thus, the younger generation tend to reduce the financial support for older adults if older adults benefit from health insurance. On the other hand, in the context of Chinese culture, people sometimes feel embarrassed about others’ gratuitous grants and regard it is a favor they owe. Thus, accepting fewer funds from others is reasonable for the Chinese older adults when they can obtain help from formal organizations.

Formal supports could directly help older adults avoid poverty by reducing their OOP costs. In the present study, holding health insurance is beneficial for forming positive formal supports, and the effect of reimbursement rate of inpatient care on formal support was the most prominent, which indicated that the key role of formal support in alleviating poverty lies in reducing medical costs and improving health as much as possible. Evidence showed that with the popularization and improvement in health insurance, the percentage of total health expenditure paid OOP in China dropped from 60% in 2001 to 40% in 2008 and then decreased to 27.65% in 2020 [57]. Moreover, financial assistance from disease insurance of commercial organizations is usually guaranteed and will be helpful for preventing the occurrence of catastrophic health expenditure (CHE) in low-income households. Xu YJ et al. also found that the presence of commercial health insurance decreased the odds of facing CHE [58], thereby effectively avoiding the risk of poverty. Finally, individuals with health insurance are more motivated to obtain health services, which contribute to prompt health recovery and reduction in high medical costs due to further aggravation of the illness. This finding is verified by our study as well as that of Gondek D et al. [59], that is, poor physical health is associated with worse economic condition, and keeping health or recovering after treatment could avoid a vicious cycle between illness and poverty [60].

The findings of this research also provide policy options and directions for the governance of relative poverty after poverty alleviation for the Chinese government. By the end of 2020, China has achieved comprehensive poverty alleviation, but poverty is dynamically changing, and poverty alleviation is not a one-off effort. Especially for the elderly group with high instability after poverty alleviation and health risks, incorporating necessary physical examinations, major disease screening and other preventive health care expenditures and outpatient expenses into the payment scope of basic medical insurance, continuously improving and optimizing the medical insurance drug catalogue, dynamically monitoring and providing medical assistance to sick older adults in time, will help achieve long-term stable poverty alleviation and consolidate the achievements of poverty alleviation.

Several limitations must be recognized. First, the analysis was based on self-reported data. The information on the income each household received or the various costs related to health services and cash received from others was probably subjected to recall or other forms of self-reported bias, which is also present in other studies that use CHARLS data and is rarely avoidable [61]. Second, individuals who were too poor to seek healthcare and thus did not incur medical expenses were possibly not captured in the analysis, thereby possibly underestimating the impact of social support on economic risk. Third, even though both formal and informal aspects of social support were considered, the multidimensional properties of support such as emotional supports and support for life care were not yet fully examined. Therefore, improving the way social support was measured is important in future research. Fourth, some confounding factors were not observed in this study, which may limit our interpretation of the association between social support and poverty. Finally, only cross-sectional data were used in our study, it is impossible to conduct time series analysis and dissecting and separating possible reciprocal effects between variables would be challenging. Thus, longitudinal population-based studies about the effects of social supports on subsequent economic condition are needed to understand the magnitude and extent of poverty alleviation resulting from social supports.

## Conclusions

Our study has revealed a dilemma in reducing the economic burden of disease and receiving family assistance for older adults, and owning a complete health security and high level of medical expense compensation would directly alleviate poverty. Thus, evidence from this study can be used to promote early intervention for older adult’s wellbeing. First, a need arises for the government to encourage primary care through a social pooling mechanism for outpatient services to redirect resources for subsidizing the less affluent in the insurance schemes and increase the percentage of insurance compensation. Second, social workers can leverage specific cultural antecedents to help ensure that older adults are supported adequately under sharing obligations across families and government at all levels. Finally, policy advocacy is needed to strengthen the management and supervision for the health insurance market and improve the role of supplementary insurance in the universal health coverage, particularly for older adults with low education, unmarried, living in rural areas, and with poor health. After all, it is the right of every individual to have equal and easy access to health and a better life.

## Data Availability

The datasets generated and analysed during the current study are available in the China Health and Retirement Longitudinal Study (CHARLS) repository, https://charls.charlsdata.com/pages/Data/2018-charls-wave4/zh-cn.html.

## Abbreviations

OOP: Out-of-pocket
UEBMI: Urban Employee Basic Medical Insurance
URRBMI: Urban and Rural Resident Basic Medical Insurance
CHARLS: China Health and Retirement Longitudinal Study
PPS: Probability proportional to size
CNY: China Yuan
RMB: Renminbi
USD: United States dollar
SEM: Structural equation model
RMSEA: Root mean square error of approximation
AIC: Akaike’s information criterion
ECVI: Expected cross-validation index
AGFI: Adjusted goodness of fit index
CFI: Comparative fit index
TLI: Tucker-Lewis index
CHE: Catastrophic health expenditure
SD: Standard deviation
OR: Odds ratio
CI: Confidence interval
